# Hiram J. Friedsam Award: Reflections of Mentors and Mentorship

**DOI:** 10.1093/geroni/igab046.547

**Published:** 2021-12-17

**Authors:** Pamela Elfenbein

**Affiliations:** University of North Georgia, Gainesville, Georgia, United States

## Abstract

Being honored with the AGHE Hiram J. Friedsam Mentorship Award brought into sharp focus the mentors who have guided my journey and a desire to share this moment with them. Preparation for this session provided the opportunity to reach out to those who have shaped my path and share memories of our journeys. Lesa Huber, when awarded the Hiram Friedsam Mentorship Award in 2013, wrote “those of us who have had great mentors know it is not so much what is said, but how it is said that is at the core of effective mentorship.” This presentation reflects on relationships built, wisdom shared, guidance offered, lessons learned by design and unintentionally, and passed on in my own way to the next generation.

